# Hybrid Tele and In-Clinic Occupation Based Intervention to Improve Women’s Daily Participation after Breast Cancer: A Pilot Randomized Controlled Trial

**DOI:** 10.3390/ijerph18115966

**Published:** 2021-06-02

**Authors:** Khawla Loubani, Rachel Kizony, Uzi Milman, Naomi Schreuer

**Affiliations:** 1 Department of Occupational Therapy, Faculty of Social Welfare & Health Sciences, University of Haifa, Mount Carmel, Haifa 31905, Israel; rkizony@univ.haifa.ac.il (R.K.); nschreuer@univ.haifa.ac.il (N.S.); 2 Clalit Health Services, Haifa and Western Galilee, Tel Aviv 62098, Israel; UZIMI@clalit.org.il; 3 Sheba Medical Center, Department of Occupational Therapy, Tel Hashomer, Ramat Gan 52621, Israel

**Keywords:** activities of daily living, breast cancer, occupational therapy, self-management, tele-rehabilitation, hybrid intervention

## Abstract

Background: Women after breast cancer (BC) cope with decreased daily participation and quality of life (QOL) due to physical, cognitive, and emotional symptoms. This study examined a hybrid occupation-based intervention, Managing Participation with Breast Cancer (MaP-BC), to improve daily participation in their meaningful activities. Methods: Thirty-five women after BC phase were randomly allocated to the MaP-BC intervention (*n* = 18) or control (*n* = 17) group (standard care only). Assessments were administered at baseline (T1), 6-week (T2), and 12-week (T3) post-T1. Main outcome: perceived performance and performance-satisfaction with meaningful activities according to the Canadian Occupational Performance Measure. Secondary outcomes: retained activity levels (Activity Card Sort), QOL (Functional Assessment of Cancer Therapy-Breast), cognitive abilities (Montreal Cognitive Assessment and Behavior Rating Inventory of Executive Function), and upper-extremity functioning (Disability of Arm, Shoulder, Hand). Results showed significant interaction (group x time) effects for the primary outcome in performance, F(2,66) = 29.54, *p* = 0.001, ɳ_P_^2^ = 0.472, and satisfaction, F(2,66) = 37.15, *p* = 0.000, ɳ_P_^2^ = 0.530. The intervention group improved more in performance, t = 5.51, *p* = 0.0001, d = 1.298, and satisfaction, t = −5.32, *p* = 0.0001, d = 1.254, than the control group between T1 and T2. Secondary outcomes demonstrated within-group improvements. Conclusion: MaP-BC, a comprehensive occupation-based hybrid intervention tailored to women’s functional daily needs after BC, improved participation in meaningful activities within a short period.

## 1. Introduction

Women after breast cancer (BC) cope with disease and treatment-related medical, physical, cognitive, and emotional symptoms [1]. These symptoms challenge women’s ability to maintain their functioning and participation in meaningful daily activities in the areas of self-care, leisure, and social activities [2,3] and affect their quality of life (QOL) [4] in the short and long terms [5]. Successfully maintaining the desired level of participation in daily activities enhances women’s general health and well-being [6]. However, physical BC-related symptoms, such as limited range of motion, cause limitations in activities that require use of the affected upper limb [7]. Moreover, limitations in or avoidance of daily activities may further increase hand-function impairments. Cognitive symptoms related to cancer and chemotherapy include reduced attention, memory, and executive functions [8]. These symptoms may challenge the performance of cognitively demanding activities, such as those involving multi-tasking or faster information processing [9]. Cognitive impairments also are associated with emotional and psychological consequences, such as anxiety and depression [10] arising from the diagnosis of cancer, its symptoms, or fear of cancer recurrence [11].

Daily functioning encompasses both physical and psychosocial abilities [12]. Living with restricted abilities for an uncertain duration affects women’s participation in meaningful activities and their QOL [13] for many years after the BC diagnosis. Therefore, women diagnosed with BC can benefit from early rehabilitation to ameliorate functional deterioration and enhance participation [4]. Maximizing daily function and participation is a unique role of occupational therapists. They specialize in evaluating and treating the client’s complex functional needs and barriers within that client’s context and environment [14]. Occupational therapy (OT) interventions have the potential to address the survivor’s current abilities and disease-related restrictions [15] to enhance participation in meaningful activities and facilitate independence and QOL [16,17]. These interventions also offer tools to improve performance by adapting daily activities and environments [5,16] while considering the need of long-term management.

Women after BC need to actively manage their BC-related long-term consequences [18]. Therefore, to increase their competence and self-efficacy, the rehabilitation process should integrate “patient centered” approaches [19] and specifically those applying centered attention to the patient by recognizing the uniqueness of the person, focusing on their capacities, and supporting their self-determination to strive to achieve their goals and to be independent [20], such as self-management approaches. This is in line with the current health system comprehensive approach to health care, that focuses on the patient’s needs aiming at increasing quality of care [21]. Indeed, studies have reported the effectiveness of OT interventions that use self-management approaches with BC patients in enhancing the participation of women undergoing chemotherapy, using telephone-based sessions [17,22] and in addressing daily challenges related to cognitive dysfunction [23]. However, randomized control trial (RCT) studies that examined similar programs are scarce.

To address these issues, we developed a comprehensive occupation-based and individually tailored hybrid intervention—Managing Participation with Breast Cancer (MaP-BC)—that focuses on improving women’s participation in meaningful daily activities in the subacute phase of BC. It uses a hybrid approach, integrating individual in-clinic and tele-rehabilitation sessions. The MaP-BC adopts the Model of Human Occupation [15] as the overarching theoretical basis to deeply understand, from an occupation-based perspective, how women after BC experience disruptions to their occupational performance and participation [24]; how they are motivated to do things, what occupational adaptation process is needed following BC diagnosis [2], and what effort is needed to regain their abilities to participate in activities that were part of their daily routines prior to BC [9]. Practically, MaP-BC integrates principals drawn from three approaches: (1) self-management strategies (i.e., solving problems, making decisions, taking action, and utilizing resources) [19]; (2) biomechanical approach (i.e., managing sensory-motor symptoms and function, saving energy, and environmental adaptations) [25]; and (3) multi-context treatment approach to train metacognitive strategies related to women’s daily activities [26]. Tele-rehabilitation incorporates the use of information and communication technologies (e.g., text messages or videoconferencing) to improve availability of rehabilitation services to clients by providing therapy beyond the physical clinical environment. Thus, tele-rehabilitation facilitates and enhances services’ accessibility in terms of time and distance; avoiding wasting time. Travel costs and risks travelling to and from the clinic [27,28]. Tele-rehabilitation can be delivered either in a synchronous (real-time) where the therapist and client are communicating online, each located in a different environment, or in an asynchronous manner, where communication is offline [29]. Tele-rehabilitation follows the same general principles of traditional rehabilitation except for methods that require “hands on” such as when facilitating movements [30]. In the Map-BC, the tele-rehabilitation sessions aimed to enhance accessibility and continuity of the rehabilitation process by taking advantage of home environments to facilitate self-management and generalize treatment gains in real life. In fact, the hybrid approach helps narrowing the gap between the clinic environment and the home environment. The hybrid approach is specifically advantageous to people with a weak immune system and low energy, who require multiple visits to clinics due to cancer-related treatments [31].

This study’s primary aim was to examine the feasibility of the hybrid MaP-BC intervention to improve participation in meaningful daily activities of women in their subacute phase after BC compared with standard care (medical and psychosocial or allied health care). The secondary aims were to examine the feasibility of the hybrid MaP-BC in improving QOL, general participation, and motor and cognitive abilities.

## 2. Materials and Methods

This study was approved by the Helsinki committee of Clalit Health Services (0239-16-COM1) and Ethics committee of the University of Haifa (No.303/19) and registered at the Israeli Health Ministry clinical trials website (MOH_2018-01-17_002105). A single-blind RCT design was used. Participants were randomly allocated to either the intervention or control group using block randomization with an allocation ratio of 1:1. Randomization was performed by a researcher that was not involved in participants’ recruitment and had no contact with any of the participants along the study and in addition, allocation was concealed. Assessments were administered prior to randomization (baseline T1), at six weeks (T2), and at 12 weeks post-baseline (T3).

### 2.1. Participants

Women were eligible for enrollment if they were insured by the largest health service in the country. Inclusion criteria were women with invasive ductal carcinoma, Stages I to III who (a) were 3 to 24 months post-BC diagnosis, (b) had completed primary adjuvant therapies, (c) were previously healthy, and (d) reported difficulties or decreased participation in daily activities compared with before BC. The exclusion criterion was severe (neurological, cognitive, mental, or orthopedic) disability that affected daily functioning. The sample size calculation resulted in 35 women according to the primary outcome measure, calculated using G * Power software [32] for repeated measures mixed design (within between interactions) to obtain a moderate effect size (v) of 0.40, power of 0.80, and α of 0.05.

### 2.2. Procedure

Women were referred to the study by staff from their oncology clinics in northern Israel. Eligible women signed the informed consent form and completed the T1 assessment. To minimize bias, the women were randomly allocated to the intervention or control groups after T1 by a researcher who was not involved in recruiting, assessing, or treating the participants. Assessments were administered by OT assessors who were blinded to the group allocations. The intervention group received the 6-week hybrid MaP-BC intervention plus standard care. The control group received standard care only. Between T2 and T3, both groups received a weekly follow-up phone call to document medical interventions and to minimize dropouts. Women in the control group were offered the hybrid MaP-BC intervention after completing T3.

#### MAP-BC Intervention

The MAP-BC is a 6-week hybrid intervention consisting of alternating weekly in-clinic OT sessions and tele-rehabilitation sessions (from the woman’s home) for 12 total sessions with a licensed OT. The hybrid MaP-BC protocol (Table A1) is tailored to the occupational needs and goals that each woman defined as important, considering her habits, roles, abilities, limitations, and environmental and life contexts (e.g., family and work). The women are trained in various strategies to achieve their specific goals related to personally meaningful activities.

The first meeting was at the OT clinic and included setting functional meaningful goals to be achieved during the following sessions, planning a timeline, and training the woman to use the computerized CogniMotion tele-system (ReAbility Online, Gertner Institute, Sheba Medical Center, Ramat Gan, Israel) [1].

The CogniMotion tele-system is a 3D video capture camera-based system (at the time of the current study with the Microsoft’s Kinect 3D sensor) to capture upper extremities movements while interacting with virtual games and tasks (e.g., con-structing a puzzle or preparing a pizza) (Description available online: https://www.reabilityonline.com/tele-motion/ (accessed on 28 May 2021)). The equipment was located in the woman’s home and connection to the OT’s computer was done via the internet. The CogniMotion’s usability, validity, as well as its feasibility and effectiveness, have been described previously [30,33]. As mentioned above, during the first meeting, the women experienced the tele-rehabilitation games and tasks, and the OT personalized the level of difficulty according to each woman’s motor and cognitive abilities as well as her functional needs. At the end of the meeting, each woman was loaned a camera to be used during the tele-rehabilitation sessions as well as for self-practice between sessions.

The following meetings included training on self-management and meta-cognitive strategies needed to manage symptoms and minimize barriers to participating in the selected meaningful activities (e.g., self-knowledge, awareness and processing strategies, using cognitive strategies such as self-talk to enhance attention, reorganizing priorities, understanding and practicing activity demands, preparing weekly action plans, and utilizing potential environmental and social resources). Depending on the woman’s functional needs and motor abilities, the sessions also covered several aspects of sensory-motor training (i.e., reducing range-of-motion limitations, strengthening the upper extremity, and improving fine-motor functioning).

During the tele-rehabilitation sessions, ways to implement the weekly plan and to transfer the strategies trained at the clinic into daily activities were discussed. These meetings enabled the women to address the dilemmas they faced during the week in coping with other daily activities. In addition, during these tele-rehabilitation online sessions, virtual games and tasks of the CogniMotion tele-system were used to enhance upper-extremity movements and cognitive abilities according to the participant’s needs. Moreover, further adjustments to the games/tasks were performed as needed and additional games/tasks were taught. The occupational therapist provided technical support, when needed.

### 2.3. Measures

The following tools were used at each assessment (T1, T2, and T3): demographic and clinical-data questionnaire that included age, education, marital status, months since diagnosis, surgery, BC stage, medical therapies upon diagnoses, and additional therapies (reported at T1 only).

#### 2.3.1. Primary Outcome

##### Canadian Occupational Performance Measure

The Canadian Occupational Performance Measure (COPM) is a semi-structured interview that measures participation in meaningful daily activities [34]. Participants choose four activities and rate them on an ordinal 10-point scale relating to perceived performance from 1 (not able to do) to 10 (able to do extremely well) and to performance satisfaction from 1 (not satisfied at all) to 10 (extremely satisfied). Final scores were mean ratings of performance and performance satisfaction of the four activities. The same activities were rated at T1, T2, and T3. In addition, we summed, the number of activities in which each woman achieved a minimal detectable improvement (≥2 points) for performance and performance satisfaction [35]. Validity and reliability of the COPM has been established in several populations [36] and used as a primary outcome in RCT studies [37].

#### 2.3.2. Secondary Outcomes

##### Activity Card Sort

The Activity Sort Card (ACS) assesses self-reported participation in 89 everyday activities divided into four domains: instrumental activities of daily living (e.g., driving, paying bills); social-cultural (e.g., traveling, visiting friends); low-demand leisure activities (e.g., watching television, reading); and high-demand leisure activities (e.g., hiking, sports) [38]. This RCT used the ACS version adapted for patients living in the community [39]. Women are asked to sort the activities they did before illness and categorize each activity into 1 (doing now), 0.5 (doing less after illness), reflecting reduced participation, or 0 (given up due to illness). Each woman’s total retained activity level in each domain was calculated (sum of the activities performed at each assessment divided by the sum of activities performed before illness). A retained activity level score of less than 100% indicates reduced participation within the comparison period (i.e., T1, T2, and T3) compared with before the illness. The ACS demonstrates good test–retest reliability and construct validity [40].

##### Disability of Arm, Shoulder, and Hand: Quick Version

The Disability of Arm, Shoulder, and Hand: Quick version (Quick-DASH) assesses self-reported disability of the upper extremities [41]. The Quick-DASH includes 11 items rated on a 5-point ordinal scale. The final score is the mean score of all items (at least 10 of 11) converted to a score ranging from 0 to 100. Higher scores indicate higher disability. The Quick-DASH has been found reliable and valid for assessing upper extremity disability after BC [42].

##### Hydraulic Hand Dynamometer

The hydraulic hand dynamometer assesses hand-grip strength [43] in kilograms and has been found highly reliable [44].

##### Montreal Cognitive Assessment

The Montreal Cognitive Assessment (MOCA) assesses general cognitive abilities in eight domains: visuospatial/executive functioning, naming, memory, attention, language, abstraction, delayed recall, and orientation [45]. Scores range from 0 to 30; a score of 26 or above reflects intact cognition. The MOCA has good internal consistency reliability [45] and has been found sensitive for measuring mild cognitive impairment in cancer survivors [46].

##### Behavior Rating Inventory of Executive Function: Adult Version

The Behavior Rating Inventory of Executive Function-adult version (BRIEF-A) is a reliable and valid comprehensive assessment of self-reported executive functions [47]. The tool includes 75 items capturing adults’ self-reported everyday executive functions (i.e., inhibit, shift, emotional control, working memory, plan/organize, task monitor, and organization of materials). Respondents answer how often each assessed behavior was a problem on a scale of 1 (never), 2 (sometimes), and 3 (often). The overall standard score (global executive composite) is transformed into t scores; higher scores reflect greater experienced difficulties. The BRIEF-A has moderate to high internal consistency and test–retest stability [47]. The current RCT used the final t scores for the global executive composite.

##### Functional Assessment of Cancer Therapy-Breast

The Functional Assessment of Cancer Therapy-Breast (FACT-B) assesses health-related QOL after BC diagnosis [48]. It includes 37 questions with responses given on a 5-point Likert scale from 0 (not at all) to 4 (very much). The questions constitute five dimensions of wellbeing: physical, social/family, emotional, functional, and additional concerns. The scores of all dimensions are summed to a total score ranging from 0 to 148, with higher scores indicating better health-related QOL [49]. This RCT used the FACT-B total and emotional well-being (six items; possible score range of 0–24) scores. The FACT-B has good reliability, validity, and internal consistencies [48].

### 2.4. Statistical Analysis

We verified normality of the data by Shapiro–Wilk tests and statistical tests performed accordingly. Scores for all randomized women were analyzed using intention-to-treat analysis [50] with Statistical Package for the Social Sciences (SPSS) version 25 (IBM Corp, Armonk, NY, USA) and independent t and Mann–Whitney tests to examine differences between groups at T1. We used repeated measures ANOVA mixed design to examine within-group (T1, T2, and T3) and between-group (intervention vs. control) differences for normally distributed primary (COPM) and secondary outcomes (ACS social-cultural and instrumental activities of daily living RALs, BRIEF-A, Quick-DASH, dynamometer, FACT-B). Partial eta square (ɳ_p_^2^) were calculated. To interpret significant main effect of time, post-hoc pairwise comparisons (between each time point) with Bonferroni correction were conducted. To interpret significant interaction effects, paired t tests (between each time point) within each group were completed (Cohen’s d effect size was calculated). For non-normally distributed outcomes (COPM number of activities improved ≥2 points, ACS high- and low-demand leisure domain, RALs, and MOCA), we performed nonparametric tests. We used Friedman tests to examine within-group (T1, T2, and T3) differences, Wilcoxon signed-rank tests to examined differences between each time point, and Mann–Whitney tests to examine between-group differences at each time point. Effect sizes for significant nonparametric tests (r^2^) were calculated [51].

## 3. Results

**Figure 1:**
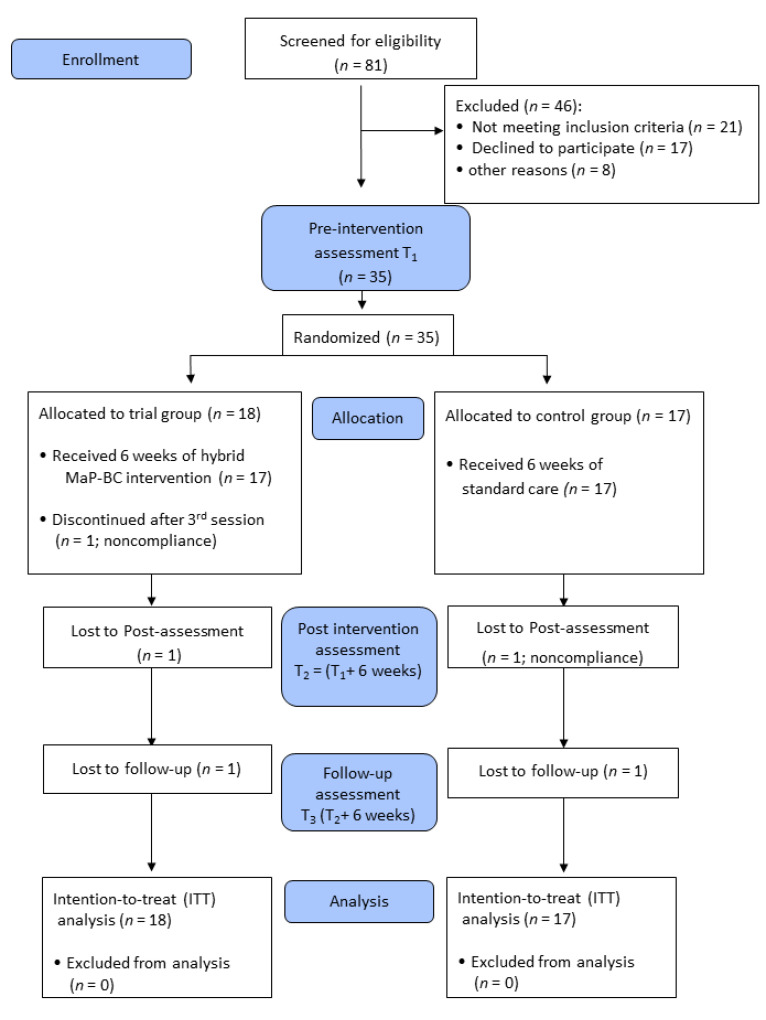
Randomized control trial CONSORT diagram of the study. Abbreviations: T1 = Time 1; T2 = Time 2; T3 = Time 3; MaP-BC = Managing Participation with Breast Cancer; ITT = Intention-To-Treat analysis.

Figure 1 presents the flow of study participants. Thirty-five women (mean age 49.97 years + 11.97) were randomly allocated to the intervention (*n* = 18) and control (*n* = 17) groups. No adverse events were reported by any of the participants.

At T1, no significant differences were found between the groups with respect to primary and secondary outcomes, demographic, and BC clinical characteristics except months since diagnoses (Table 1).

### 3.1. Primary Outcome

#### Participation in Meaningful Daily Activities (COPM)

Participants set a large variety of functional goals. Return to physical activity (sport, walking) was one of the most frequent goals in both groups (intervention, 12 women (67%); control, 13 women (76%)). Significant main effects of time and group by time interaction effects were found for performance and performance satisfaction (Table 2). Pairwise comparisons showed that the improvement over time was significantly different only between T1 and T2 for performance (*p* = 0.0001) and performance satisfaction (*p* = 0.0001). Paired t tests showed that the intervention group had greater improvement between T1 and T2 in performance (t = 5.51, *p* = 0.0001, d = 1.298) and satisfaction (t = −5.32, *p* = 0.0001, d = 1.254) compared to the control group (performance: t = −2.38, *p* = 0.03, d = 0.578; satisfaction: t = −3.87, *p* = 0.001, d = 0.939).

Additionally, a significant between-groups difference (z = −2.012, *p* = 0.044) with moderate effect size (r^2^ = 0.225) was found in the number of meaningful activities with significant detectable improvement (>2 points) in performance between T1 and T2 (intervention group: median = 2, interquartile range = 1–4; control group: median = 1, interquartile range = 0–2).

### 3.2. Secondary Outcomes

Within- and between-group comparisons of parametric tests are presented in Table 2. In general, pairwise comparisons showed significant differences only between T1 and T2 for QOL (FACT-B; *p* = 0.005), self-reported executive functioning (BRIEF-A; *p* = 0.001), and upper-extremity functioning (Quick-DASH; *p* = 0.006). Within-group comparisons of nonparametric tests are presented in Table 3. No significant differences were found between the groups for participation in high-demand leisure activities at T3 (z = −1. 61, *p* = 0.109) or for cognitive abilities (MOCA) at T2 (z = −1.12, *p* = 0.262) and at T3 (z = −1.51, *p* = 0.130).

## 4. Discussion

This study examined the hybrid MaP-BC, an individualized occupation-based intervention to improve women’s daily participation in the subacute phase after BC diagnosis. The high retention and compliance rates indicate that the hybrid in-clinic and tele-rehabilitation intervention was feasible. The results show greater improvement in the intervention group than in the control group in a relatively short time and mainly in the primary outcome of regaining performance of meaningful activities. In addition, within-group analyses reveal improvement in regaining participation in high-physical demand activities and cognitive abilities.

The improvement found in women’s participation in meaningful activities indicates that the hybrid MaP-BC intervention initiated the women’s process of adaptation to their new situation by providing them with strategies and tools that address their individual needs [5]. These findings are similar to other studies that used the COPM as an outcome measure among women with BC in a single-arm study design [23]. However, by using an RCT design, the current study strengthens the impact of the intervention by showing greater improvements in mean scores of performance and satisfaction, as well as meaningful clinical change.

Women in the current study prioritized high-demand leisure activities (e.g., sports and walking) as most meaningful, in line with Lyons et al.’s [22] findings. Notably, improvement in this domain was significant only within the intervention group. Participation in meaningful activities [5], and specifically in physical activities [53], enhances women’s general health and well-being. As such, these findings emphasize the importance and timing of the hybrid MaP-BC intervention and its added value compared with the standard care that the control group received. Generally, women are considered “healthy” after medical treatment and expected to resume their participation as before BC. In fact, they struggle with daily difficulties and need professional support to return to their previous roles and daily routines [18,22,24].

The novelty of the hybrid MaP-BC lies in its integration of theoretical and practical rehabilitation approaches, in addition to in-clinic and tele-rehabilitation sessions tailored to the women’s functional daily needs and life contexts. The self-management approach [19] enables women to share their concerns regarding their participation. Its strategy of “gaining knowledge” [54] raises women’s awareness and improves their understanding of how body impairments and symptoms (i.e., fatigue, reduced strength, and attention deficits) affect their performance and participation in meaningful activities. Women are encouraged to use “decision-making” strategies and suggest possible solutions to manage the effects of symptoms and body limitations, as well as use environmental adaptations and support resources to optimize their daily functioning. The cognitive and metacognitive approaches provide strategies [26] to overcome attention and memory difficulties interfering with daily functioning and life contexts. The biomechanical-rehabilitation approach addresses body impairments and limitations and focuses on performing daily activities. Biomechanical and ergonomic principles have been used to make environmental adaptations and enhance appropriate use of the body [25]. The tele-rehabilitation sessions contributed to the accessibility and flexibility of the intervention, as shown in other studies [17,31].

The in-person communication in the clinical context enabled establishing therapeutic alliance and using diverse tools. The alternate tele-rehabilitation sessions addressed women’s dilemmas around coping with other daily activities that arose during the week and encouraged them to train the different strategies and capacities in their natural context. The combined sessions improved the feasibility of the MaP-BC intervention, leading to improvement among the intervention group after a relatively short period of time. The high compliance could be attributed to the use of tele-rehabilitation sessions that enabled women to receive more treatment sessions without the need to spend time traveling to the clinic and therefore could better fit in their busy schedule and accommodate their residual symptoms such as fatigue and low endurance.

Women were highly engaged in the intervention and benefited from it. In addition, informal communication with the women indicated their satisfaction with its accessibility and with it being tailoring to their individual needs after BC diagnosis. For example, during the hybrid MaP-BC sessions, the women were encouraged to plan a weekly balanced schedule using strategies such as prioritizing between activities to save energy, making checklists to address attention and memory complaints, adapting the technology and environment, and applying to informal and formal support at home and at work.

In this study, both groups demonstrated improvements in secondary outcome measures (QOL, executive functioning, and upper-extremity functioning). This is consistent with studies showing that participation in RCTs contributes to outcome improvements regardless of whether the participants received the intervention [55]. The fact that women from the control group knew that they would be offered the hybrid MaP-BC intervention later may have contributed to higher motivation and retention. Moreover, this effect was enhanced by using the comprehensive occupation-based assessment approach, which includes semi-structured interviews that query about meaningful activities and life goals and self-reported questionnaires regarding the impact of physical (Quick-DASH), cognitive (BRIEF-A), and emotional (FACT-B) symptoms on daily functioning and quality of life. This may have raised the women’s awareness of their own functional needs and barriers they wish to reduce.

### 4.1. Study Limitations

The current RCT had a small sample and therefore may have been underpowered to detect all expected differences. In terms of external validity, the study was conducted in the largest health service provider in a specific geographic area. In addition, participants included women who were diagnosed with BC for the first time and who had no other diagnoses. Women’s satisfaction with the intervention was based on their self-reports during conversations and not assessed systematically with a questionnaire or structured interview. Future studies should explore satisfaction from each part of the intervention separately, including specific questions to gain better understanding of the utility of the hybrid intervention. In addition, the current study did not relate to specific treatments such as PARP inhibitors as well as genetic counselling and testing that may affect symptoms, level of stress, and daily participation. Future studies should examine how such specific medical treatments are associated with benefits from rehabilitation interventions. Moreover, as socio-economic status may influence intervention outcomes [56], future studies should add socioeconomic parameters to control their effect. We also recommend adjusting interventions such as MaP-BC to address low socioeconomic populations and to evaluate its feasibility in those populations.

### 4.2. Study Implications

Providing individualized and occupation-based rehabilitation interventions as early as possible after cessation of breast cancer medical treatments enhance women’s daily participation. Specifically, clinicians should be aware and facilitate a variety of self-management skills (e.g., e-health strategies) as early as possible in the rehabilitation process after BC diagnosis, to enhance flexible use of these skills. Integrating tele-rehabilitation enables continuation of the rehabilitation process while considering the consequences of the women’s health conditions and contributing to the feasibility of the rehabilitation process. The use of an RCT design that is considered the highest level of evidence [55] contributes to the evidence-based-practice of OT with women coping with BC consequences. The authors will be more than happy to share more details regarding the hybrid MaP-BC intervention.

## 5. Conclusions

Women with BC need professional rehabilitation support to navigate the complexity of returning to meaningful participation after completing initial medical care. The occupation-based approach used in the current study encourages the women to overcome barriers related to fear and concerns regarding their abilities, as well as objective declines in capacities to perform daily activities. This study’s results show that a comprehensive occupation-based evaluation also may contribute to women’s ability to improve their participation. Nevertheless, the hybrid MaP-BC intervention—tailored to women’s functional daily needs and life contexts—led to improved participation within a short time. Providing rehabilitation as early as possible in the subacute phase of BC is necessary, and future studies should examine the impact of a multidisciplinary rehabilitation program on long-term daily participation of women with BC.

## Figures and Tables

**Table 1:** Descriptive statistics and comparison of demographic and breast cancer clinical characteristics (*n* = 35).

Variable	Intervention Group (*n* = 18)		Control Group (*n* = 17)		Between-Group Comparison	
	***M* (*SD*)**	**Range**	***M* (*SD*)**	**Range**	***t***	***df***
Age (years)	48.00 (11.14)	28–69	52.06 (12.80)	31–64	−1.00	33.0
Education (years)	15.06 (1.92)	12–17	13.94 (2.28)	11–17	1.57	33.0
Months since diagnosis	14.56 (5.54)	5–25	11.24 (3.17)	6–17	2.19 *	27.3
	***n* (%)**		***n* (%)**		**χ^2^**	***df***
Marital status						
Married	11 (61.10)		12 (70.60)		0.35	1.0
Other	7 (38.90)		5 (34.40)			
BC stage						
1	2 (11.10)		6 (35.30)		3.64	2.0
2	10 (55.60)		5 (29.40)			
3	6 (33.30)		6 (35.30)			
Surgery						
Lumpectomy	10 (55.60)		13 (76.50)		1.70	1.0
Mastectomy	8 (44.40)		4 (23.50)			
Medical therapy upon diagnosis						
Chemo	14 (77.80)		11 (64.70)		0.73	1.0
Radio	13 (72.20)		12 (75.00)		0.03	1.0
Hormonal	10 (55.60)		13 (81.30)		2.56	1.0
Additional therapy						
Lymphatic	5 (27.80)		4 (23.50)		0.24	2.0
None	6 (33.30)		7 (41.20)			
Other	7 (38.90)		6 (35.30)			

* *p* = 0.037.

**Table 2:** Descriptive statistics and results of within- and between-parametric group comparisons at T1, T2, and T3.

Measure	Intervention Group (*n* = 18) *M* (*SD*)			Control Group (*n* = 17) *M* (*SD*)			Group Effect			Time Effect			Interaction Effect		
	T1	T2	T3	T1	T2	T3	*F*(*df*) (1,33)	*p*	ηp^2^	*F*(*df*) (2,66)	*p*	ηp^2^	*F*(*df*) (2,66)	*p*	ηp^2^
COPM															
PCOPM	3.83 (1.60)	6.01 (1.84)	6.15 (1.84)	4.01 (1.76)	4.90 (1.85)	5.35 (1.91)	1.18	0.286	0.034	**29.54**	0.0001	0.472	**3.51**	0.036	0.096
SCOPM	2.63 (1.61)	5.88 (2.64)	6.04 (2.45)	3.34 (1.58)	4.84 (2.11)	5.12 (2.41)	0.44	0.511	0.013	**37.15**	0.0001	0.530	**4.30**	0.018	0.115
ACS RAL															
Total	0.69 (0.18)	0.73 (0.20)	0.74 (0.20)	0.58 (0.20)	0.61 (0.20)	0.59 (0.18)	**4.61**	0.039	0.123	1.34	0.269	0.039	0.190	0.827	0.006
IADL	0.68 (0.20)	0.74 (0.23)	0.72 (0.23)	0.60 (0.22)	0.62 (0.23)	0.63 (0.20)	2.10	0.157	0.060	1.36	0.264	0.040	0.41	0.666	0.012
Social	0.65 (0.22)	0.73 (0.24)	0.69 (0.24)	0.53 (0.28)	0.56 (0.28)	0.47 (0.23)	**5.43**	0.026	0.141	1.88	0.161	0.054	0.96	0.388	0.028
Motor performance															
DASH	48.19 (20.25)	37.67 (22.13)	34.72 (21.26)	49.19 (20.58)	44.02 (22.93)	44.57 (22.73)	0.70	0.407	0.021	9.28	0.0001	0.219	1.91	0.157	0.055
Grip	17.95 (5.98)	18.99 (4.93)	20.20 (5.63)	16.61 (5.39)	17.33 (5.79)	16.73 (5.78)	1.54	0.224	0.044	1.86	0.165	0.053	1.62	0.206	0.047
Cognitive (BRIEF-A)															
GEC	64.33 (12.68)	58.44 (11.34)	58.44 (12.56)	62.18 (12.19	60.00 (8.67)	57.64 (10.80)	0.02	0.893	0.001	**8.01**	**0.001**	0.195	0.95	0.394	0.028
BRI	58.17 (12.87)	54.39 (12.17)	54.94 (13.45)	58.35 (12.68)	56.12 (9.16)	53.41 (10.02)	0.02	0.900	0.0001	**3.60**	**0.033**	0.098	0.32	0.724	0.010
MI	63.28 (12.81)	56.94 (10.60)	56.50 (11.46)	59.71 (12.60)	57.94 (9.11)	55.41 (9.83)	0.12	0.727	0.004	**9.76**	**0.0001**	0.228	1.55	0.219	0.045
FACT-B															
Total	91.55 (20.11)	99.12 (21.24)	100.75 (23.05)	89.13 (14.82)	94.99 (16.28)	95.31 (16.83)	0.44	0.510	0.0130	**8.54**	**0.001**	0.205	0.28	0.758	0.008
EWB	15.89 (4.31)	17.11 (4.78)	17.22 (4.60)	16.06 (4.02)	17.18 (5.02)	16.82 (4.60)	0.00	0.969	0.0000	2.28	0.110	0.065	1.30	0.882	0.004

Partial eta square (η_p_^2^) was calculated as effect size: small (0.02–0.13), moderate (0.13–0.26), large (>0.26) [52]. Statistically significant values in bold font. Abbreviations: ACS RAL = Activity Card Sort, retained activity level; BRIEF-A = Behavior Rating Inventory of Executive Function-adult version; COPM = Canadian Occupational Performance Measure; EWB = emotional well-being; FACT-B = Functional Assessment of Cancer Therapy-Breast; GEC = BRIEF-A global executive composite; IADL = instrumental activities of daily living; Quick-DASH = quick version of the Disability of Arm, Shoulder, Hand; SCOPM = COPM performance satisfaction.

**Table 3:** Descriptive statistics and results of within-nonparametric group comparisons.

Measure	Median (Interquartile Range)			Freidman Test			
	T1	T2	T3	χ^2^ (df = 2) (*p*)	T1–T2 (*p*)	T2–T3 (*p*)	T1–T3 (*p*)
Intervention group (*n* = 18)							
ACS (high-leisure RAL)	0.50 (0.32–0.79)	0.67 (0.31–1.03)	0.70 (0.38–1.00)	8.58 (0.014)	−0.53 (0.593)	−1.90 (0.060)	2.45 (0.014)
ACS (low-leisure RAL)	0.81 (0.66–1.05)	0.78 (0.63–1.03)	0.82 (0.58–1.07)	1.05 (0.591)	−0.31 (0.753)	−0.57 (0.570)	−0.16 (0.875)
Cognitive performance capacity (MOCA)	27.00 (25.00–28.00)	27.50 (26.75–28.25)	29.00 (27.75–29.25)	11.68 (0.003)	−1.38 (0.166)	−2.10 (0.036)	−2.89 (0.004)
Control group (*n* = 17)							
ACS (high-leisure RAL)	0.50 (0.18–0.65)	0.42 (0.11–0.88)	0.50 (0.18–0.81)	1.45 (0.484)	−1.02 (0.310)	−0.20 (0.838)	−1.13 (0.258)
ACS (low-leisure RAL)	0.75 (0.55–0.95)	0.75 (0.63–1.01)	0.75 (0.61–1.00)	2.26 (0.323)	−1.38 (0.167)	−0.16 (0.875)	−1.10 (0.279)
Cognitive performance capacity (MOCA)	26.00 (23.00–27.50)	27.00 (24.50–28.50)	26.00 (26.00–29.50)	5.70 (0.058)	−1.91 (0.056)	−0.07 (0.944)	−1.56 (0.118)

Abbreviations: ACS RAL = Activity Card Sort, retained activity level; MOCA = Montreal Cognitive Assessment.

## Data Availability

The data presented in this study are available on request from the corresponding author. The data are not publicly available due to issues of privacy.

## Appendix A

**Table A1:** Managing Participation with Breast Cancer (MaP-BC); hybrid intervention protocol.

Week	Session Type	Goals	Content and Examples
1	Clinic	Present intervention contents and principals. Set woman’s meaningful functional goals. Understand post-BC women’s priorities, habits, performance, and participation. Initial training on tele-program.	Plan dates for in-clinic and tele-rehabilitation sessions. Understand how symptoms affect woman’s performance and participation in general and specifically in the meaningful goals. Focus on one prioritized goal to attain during the first weeks. Train to use computerized tele-system during the tele-rehabilitation sessions.
	Tele	Install and starting training with the tele-system.	Train motor and cognitive performance capacities.
2	Clinic	Present self-management (SM) strategies. Examples for training SM strategies from woman’s daily life (identify/solve problems, make decisions, use resources, set action plan). Train motor/cognitive performance capacities related to performing meaningful selected activities. Set action plan for current week.	Understand which strategies the woman uses to deal with symptoms and their effects on her daily functioning. How does she use these strategies? (examples from her day-to-day life). Use weekly calendar to plan steps to attain one or two meaningful goals (when, how much, where.)
	Tele	Train motor/cognitive performance capacities related to performing selected meaningful activities. Discuss ways to transfer strategies trained at the clinic into additional daily activities at home/work/community environment.	Gradually add additional games/tasks to exercise and raise difficulty level according to the woman’s progress.
3–5	Clinic	Ongoing sessions that aim to increase sense of occupational competence through gradual achievement of the selected goals. Set action plan for the next week.	Maintain the retained goals. Progress to higher performance levels in same goal. Add goals.
	Tele	Discuss ways to transfer strategies trained at the clinic into additional daily activities at home/work/community environment.	Check if the strategies are useful. If not, decide about other ways or strategies to maintain specific activity performance.
6	Tele	Train motor/cognitive performance capacities related to performing selected meaningful activities.	
	Clinic	Discuss ways and strategies to maintain functional goals and participation. Summarize intervention process.	How to maintain the improvement? Summarize strategies used and found effective. Give summary of the strategies to the woman.
